# Characterization of the bacterial communities of psyllids associated with Rutaceae in Bhutan by high throughput sequencing

**DOI:** 10.1186/s12866-020-01895-4

**Published:** 2020-07-20

**Authors:** Jennifer L. Morrow, Namgay Om, George A. C. Beattie, Grant A. Chambers, Nerida J. Donovan, Lia W. Liefting, Markus Riegler, Paul Holford

**Affiliations:** 1 Western Sydney University, Hawkesbury Institute for the Environment, LB 1797, Penrith, NSW 2752 Australia; 2 Western Sydney University, School of Science, LB 1797, Penrith, NSW 2752 Australia; 3 National Plant Protection Centre, Department of Agriculture, Ministry of Agriculture & Forests, P.O. Box 670, Thimphu, Bhutan; 4 NSW Department of Primary Industries, Elizabeth Macarthur Agricultural Institute Woodbridge Rd, Menangle, NSW 2568 Australia; 5 Plant Health and Environment Laboratory, Ministry for Primary Industries, P.O. Box 2095, Auckland, 1140 New Zealand

**Keywords:** Endosymbiont, Microbiome, Psyllid, ‘*Ca*. Liberibacter’, *Wolbachia*

## Abstract

**Background:**

Several plant-pathogenic bacteria are transmitted by insect vector species that often also act as hosts. In this interface, these bacteria encounter plant endophytic, insect endosymbiotic and other microbes. Here, we used high throughput sequencing to examine the bacterial communities of five different psyllids associated with citrus and related plants of Rutaceae in Bhutan: *Diaphorina citri*, *Diaphorina communis*, *Cornopsylla rotundiconis*, *Cacopsylla heterogena* and an unidentified *Cacopsylla* sp.

**Results:**

The microbiomes of the psyllids largely comprised their obligate P-endosymbiont ‘*Candidatus* Carsonella ruddii’, and one or two S-endosymbionts that are fixed and specific to each lineage. In addition, all contained *Wolbachia* strains; the Bhutanese accessions of *D. citri* were dominated by a *Wolbachia* strain first found in American isolates of *D. citri*, while *D. communis* accessions were dominated by the *Wolbachia* strain, *w*Di, first detected in *D. citri* from China. The S-endosymbionts from the five psyllids grouped with those from other psyllid taxa; all *D. citri* and *D. communis* individuals contained sequences matching ‘*Candidatus* Profftella armatura’ that has previously only been reported from other *Diaphorina* species, and the remaining psyllid species contained OTUs related to unclassified Enterobacteriaceae. The plant pathogenic ‘*Candidatus* Liberibacter asiaticus’ was found in *D. citri* but not in *D. communis.* Furthermore, an unidentified ‘*Candidatus* Liberibacter sp.’ occurred at low abundance in both *Co. rotundiconis* and the unidentified *Cacopsylla* sp. sampled from *Zanthoxylum* sp.; the status of this new liberibacter as a plant pathogen and its potential plant hosts are currently unknown. The bacterial communities of *Co. rotundiconis* also contained a range of OTUs with similarities to bacteria previously found in samples taken from various environmental sources.

**Conclusions:**

The bacterial microbiota detected in these Bhutanese psyllids support the trends that have been seen in previous studies: psyllids have microbiomes largely comprising their obligate P-endosymbiont and one or two S-endosymbionts. In addition, the association with plant pathogens has been demonstrated, with the detection of liberibacters in a known host, *D. citri*, and identification of a putative new species of liberibacter in *Co. rotundiconis* and *Cacopsylla* sp.

## Background

Within the Psylloidae, three of the families proposed by Percy et al. [1], the Liviidae, Psyllidae and Triozidae, contain vectors of plant pathogenic bacteria. Vectors of liberibacters (Alphaproteobacteria) include species of *Arytainilla, Bactericera, Cacospylla, Diaphorina* and *Trioza* [[2] and references within]. Species of *Cacopsylla* transmit a range of phytoplasmas [3–8], and *Bactericera trigonica* Hodkinson vectors a phytoplasma to carrots [9]. Sometimes, individual psyllid species are able to vector a range of pathogens. Both *Trioza erytreae* Del Guercio (Triozidae) and *Diaphorina citri* Kuwayama (Liviidae) can transmit ‘*Ca.* Liberibacter asiaticus’ (hereafter CLas) and ‘*Ca*. L. africanus’ [10–12] and *D. citri* is also a vector for ‘*Ca*. L. americanus’ [13] and ‘*Ca.* Phytoplasma aurantifolia’ causing witches’ broom disease of lime [14].

These phytoplasmas and liberibacters are members of lineages that are ecologically specialized. Within their plant hosts, they are intracellular and restricted to the phloem, and within their insect hosts and vectors, they colonize various tissues and persist throughout the insects’ lifespan; hence, the insects are considered as alternative hosts rather than passive carriers [15, 16]. In addition to these two lineages, the bacterium, *Erwinia amylovora* (Burrill) Winslow et al. (Enterobacteriaceae), the causal agent of fireblight, has been found to be transmitted by *Cacosylla pyricola* (Förster) [17, 18] and, in South America, *Russelliana solanicola* Tuthill (Psyllidae) has been identified as the first known psyllid vector of a plant virus, the potato rugose stunting virus [19].

In addition to vectoring plant pathogens, psyllids, like other insects, harbour a diverse array of other bacteria [20] whose association with their host varies on a continuum from obligate to facultative [21]. The nitrogen content of phloem on which psyllids feed is low [22] particularly in concentrations of essential amino acids [23, 24]. To overcome these deficiencies, some insects have developed obligate associations with endosymbiotic bacteria (primary endosymbionts (P-endosymbionts)) [25] thereby allowing them to utilise nutrient-poor environments such as xylem and phloem [26, 27]. In psyllids, the P-endosymbiont is ‘*Ca.* Carsonella ruddii*’* (hereafter *Carsonella*; Gammaproteobacteria) that is housed within specialised host cells called bacteriocytes that aggregate into bacteriomes [28–30]. These P-endosymbionts are vertically transmitted resulting in strict co-speciation with their psyllid hosts within families, genera and species [28–30]. The resulting clonality has induced a drastic reduction in genome size, through loss of up to 90% of ancestral genes including, in some cases, the loss of some genes or gene pathways involved in the production of essential amino acids [31].

Other endosymbiotic bacteria, secondary endosymbiont (S-endosymbionts), may be facultative or obligate, and a diverse group of S-endosymbionts, predominantly from the Enterobacteriaceae, are associated with psyllid species [32]. These may have positive or negative effects on the biology of their host, but unlike P-endosymbionts, do not show uniform signs of co-speciation with their hosts [29, 33]. Some P-endosymbionts have lost the ability to synthesise certain essential amino acids, and their synthesis has been subsumed by S-endosymbionts [30, 34, 35]. In addition to enabling feeding on nutrient poor food sources, S-endosymbionts may also have the potential to affect host plant range [30, 36]. Other benefits conferred by S-endosymbionts include resistance to specific biotic or abiotic stresses. For example, the pea aphid (*Acyrthosiphon pisum* Harris) (Aphididae) is protected from the hymenopteran parasitoid, *Aphidius ervi* Haliday (Braconidae) by two S-endosymbionts [37], from the entomopathogen, *Pandora neoaphidis* (Remaudière & Hennebert) by *Regiella insecticola* Moran et al. [38] and from heat stress by an S-endosymbiont belonging to the Gammaproteobacteria [39]. Another endosymbiont that can be found within the bacteriome and other tissues is *Wolbachia* [40]. This Alphaproteobacterium can be beneficial, and some strains have been shown to supply B vitamins to their hosts [41]. However, in many species it affects reproduction and can also interfere with the transmission of pathogens [42, 43].

In Bhutan, five species of psyllids have been found feeding on Rutaceae: *D. citri*, *Diaphorina communis* Mathur, *Cornopsylla rotundiconi*s Lou, Li, Li & Cai, *Cacopsylla heterogena* Li and an unidentified species of *Cacopsylla* [44]. *Diaphorina communis* and *Ca*. *heterogena* [44, 45] and *Ca*. *citrisuga* (Yang & Li) have been shown to harbour CLas, and *Ca*. *citrisuga* (Yang & Li) can both acquire and transmit CLas [46, 47]. The microbiota of *D. citri* has been widely studied due to its ability to transmit the liberibacters associated with citrus, but little is known of the bacterial communities associated with *Cacopsylla* and *Cornopsylla* species. Given the potential of psyllids of these genera to harbour plant pathogens, for their host plants to also harbour plant pathogens [48], and for endosymbionts to be manipulated to manage vector-borne diseases [49–51], there is a need to examine their microbiomes. Therefore, this study has used high throughput 16S rRNA gene amplicon sequencing to examine the microbiome of these five species to: (1) identify the secondary endosymbionts and any other facultative endosymbionts in these psyllids; (2) to determine if these individuals carry plant pathogens such as liberibacters that are at low titre or novel and therefore difficult to detect by conventional screening.

## Results

### Psyllid microbiome analysis using 16S rRNA amplicon sequencing

A total of 48 individuals from five species were subject to 16S rRNA gene amplicon sequencing, and generated a total of 1,695,018 paired-end reads. After trimming, 92.9–96.7% of paired reads were joined, filtered (phred threshold > 19), and sequences were clustered into OTUs at 97% identity; OTUs comprising 2 or fewer reads were removed. For the remaining OTUs, 95 chimeras were identified and removed, leaving the total number of sequence reads per sample ranging from 5612 to 44,381, that clustered into 587 OTUs (Supplementary Table 2). Representative OTU sequence size ranged from 303 bp to 445 bp (mean +/− sd = 411.65 +/− 19.81). Rarefaction curves of observed OTUs per sample (Fig. S1) showed that read coverage was nearly complete. The Good’s coverage metric (Good’s coverage > 99.3%, mean 99.8%; Supplementary Table 4) showed that normalisation of read number to 5612 was sufficient for all samples, and based on rarefaction to 5612 sequences per sample (i.e., the sequence read number of the library with the overall lowest read number), collectively 416 OTUs remained, including 226 OTUs that were now only represented by a single read in the entire rarefied dataset (Supplementary Tables 3 & 4). All measures of alpha diversity showed low species richness with 9–60 detected OTUs (Supplementary Table 2), with individual psyllids only containing 2–6 OTUs at above 1% abundance.

### Identity of primary and secondary endosymbionts

**Fig. 1:**
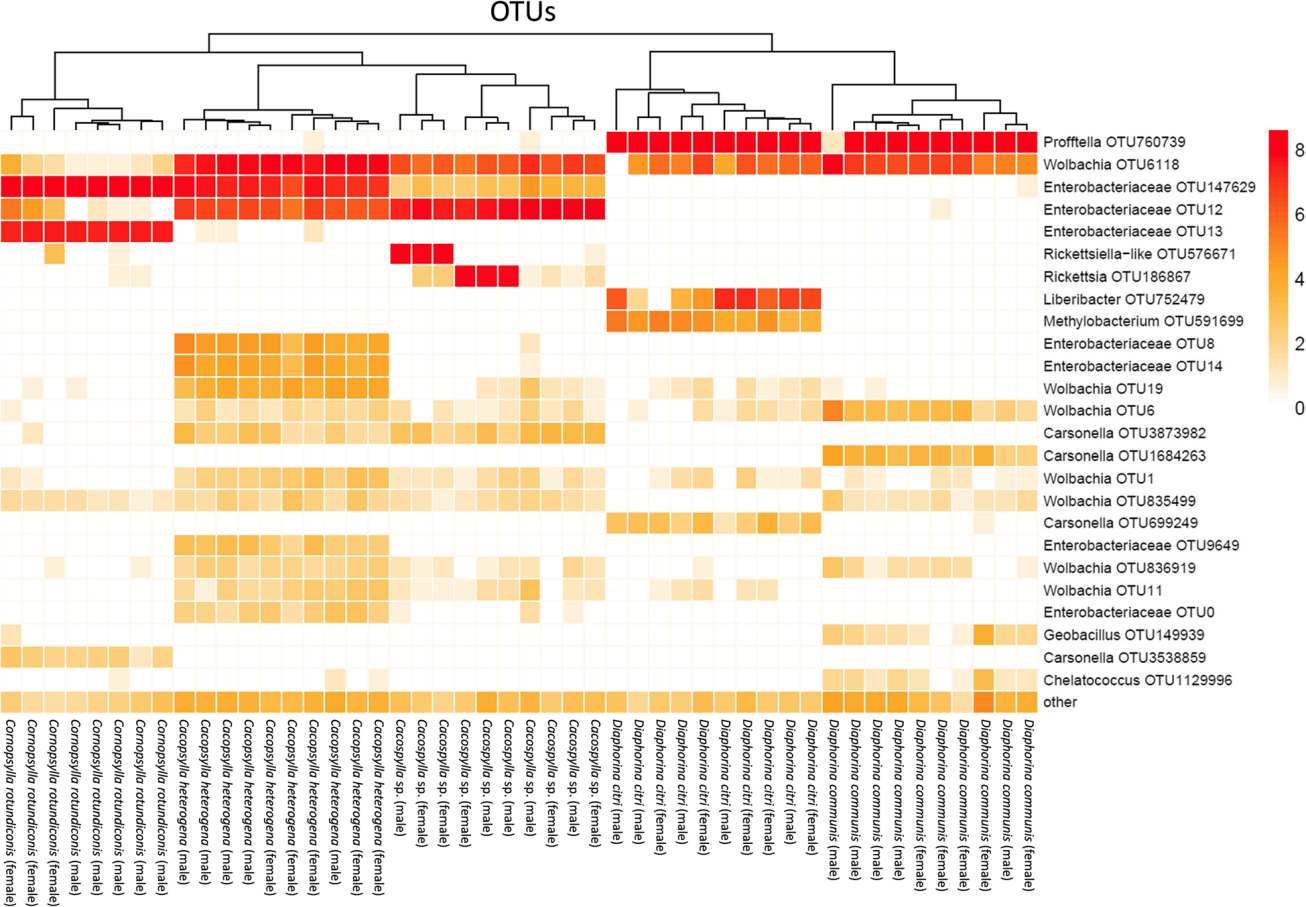
Heatmap depicting abundance of OTUs within male and female psyllids of the five Bhutanese species. The heatmap was constructed using normalized log10 abundance of each OTU in each individual

**Fig. 2:**
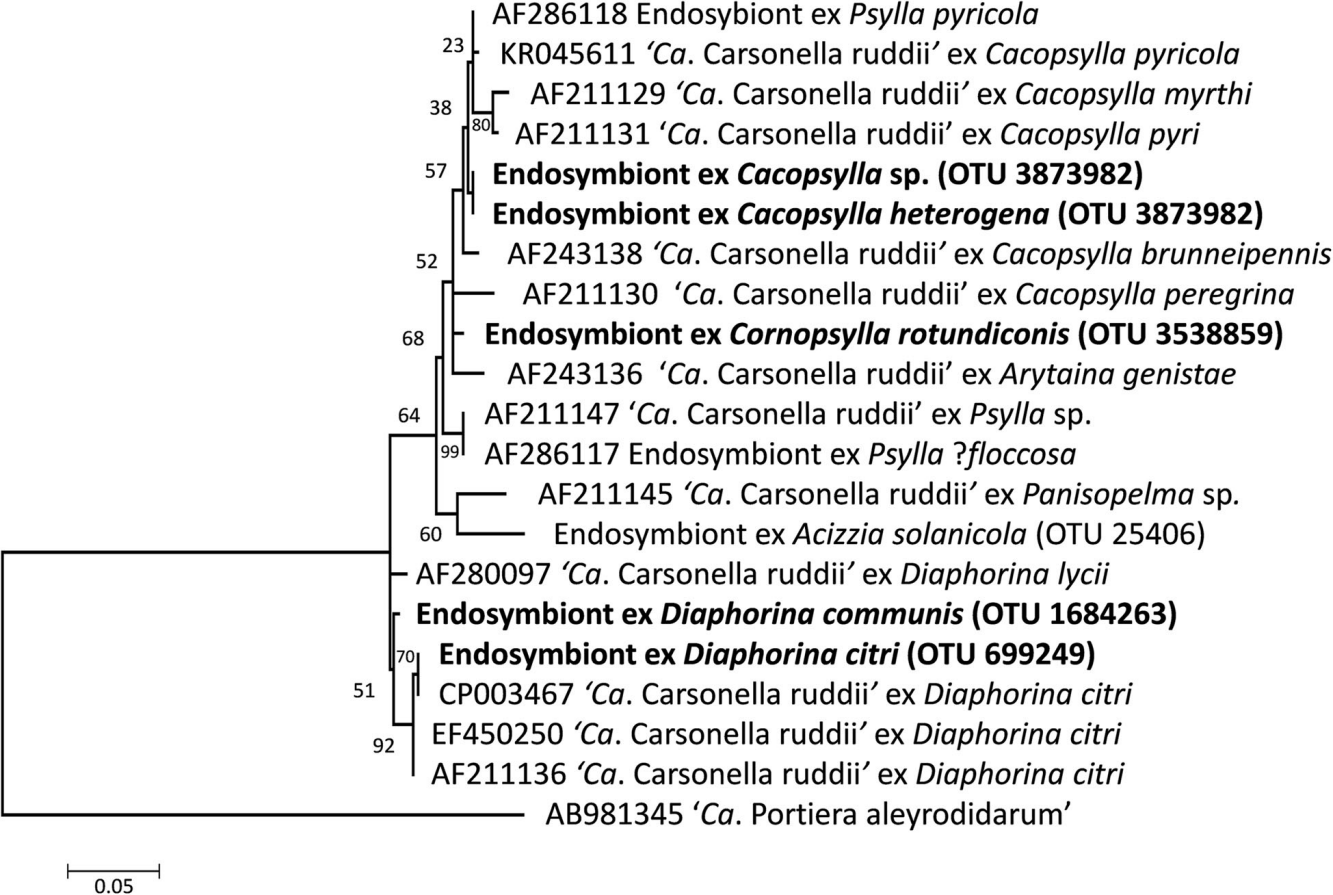
Phylogenetic analysis of 16S rRNA gene sequences of potential P-endosymbionts of the five species of Bhutanese psyllids (emboldened). The evolutionary history was inferred using maximum likelihood based on the Hasegawa-Kishino-Yano model [52]. The tree with the highest log likelihood (− 1375.27) is shown. The percentage of trees in which the associated taxa clustered together is shown next to the branches. A discrete Gamma distribution was used to model evolutionary rate differences among sites (5 categories (+*G*, parameter = 0.2691)). The tree is drawn to scale, with branch lengths measured in the number of substitutions per site. The tree is rooted with ‘*Ca*. Portiera aleyrodidarum’. (NB OTU numbers in bold classified according to Greengenes database, see Supplementary Table 3; OTU 25406 described in [53])

**Fig. 3:**
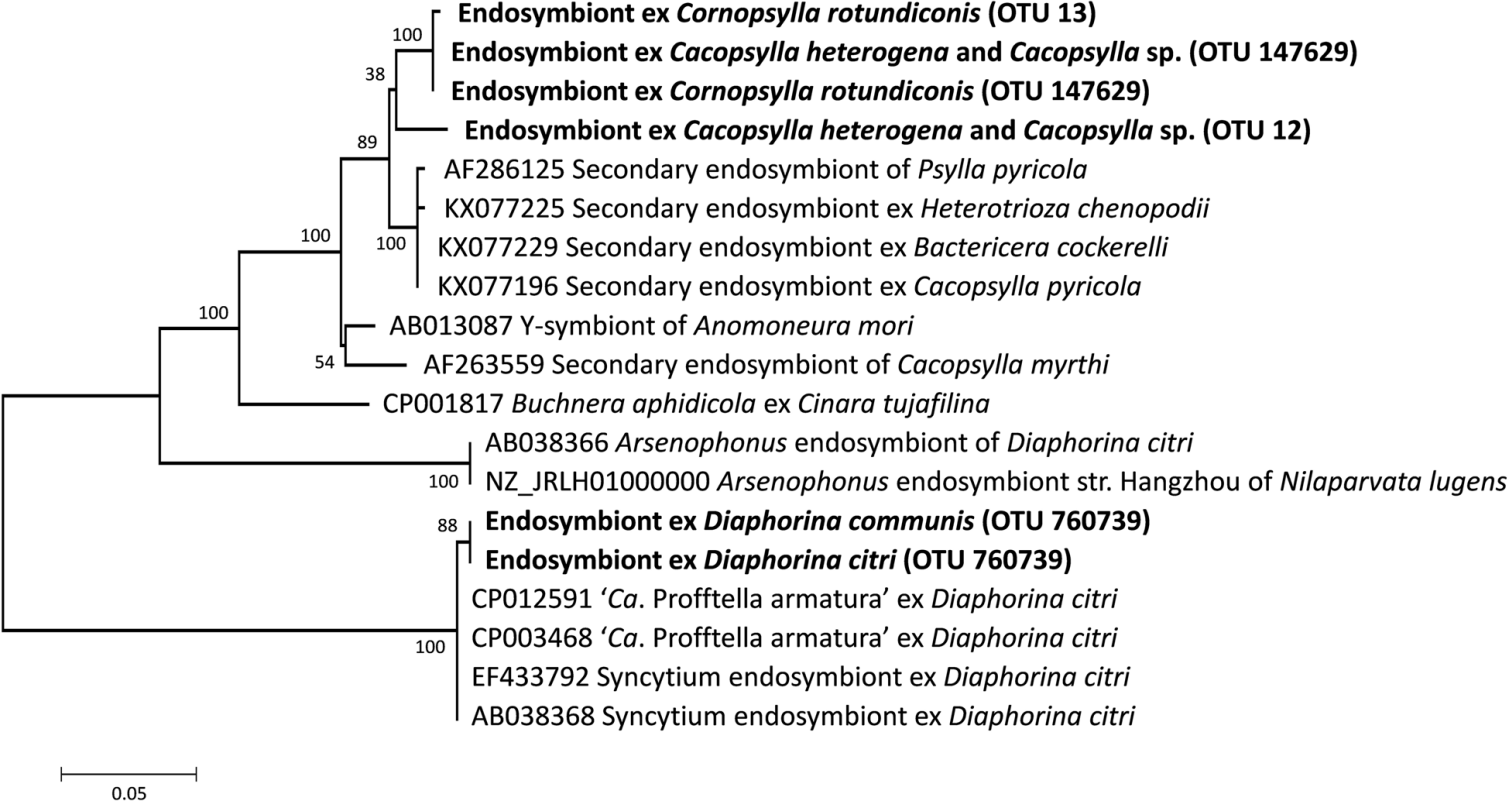
Phylogenetic analysis of 16S rRNA gene sequences of potential S-endosymbionts of the five species of Bhutanese psyllids (emboldened). The evolutionary history was inferred using maximum likelihood based on the Kimura 2-parameter model [54]. The tree with the highest log likelihood (− 1451.55) is shown. The percentage of trees in which the associated taxa clustered together is shown next to the branches. The rate variation model allowed for some sites to be evolutionarily invariable ([+I], 35.53% sites). The tree is drawn to scale, with branch lengths measured in the number of substitutions per site. The tree is unrooted. (NB OTU numbers classified according to Greengenes database; see Supplementary Table 3)

All psyllid individuals contained *Carsonella* (Figs. 1 & 2), the primary endosymbiont of psyllid species. Four OTUs were classified as *Carsonella*; *D. citri*, *D. communis* and *Co. rotundiconis* possessed one *Carsonella* strain each that was fixed in that species, and *Ca*. *heterogena* and the unclassified *Cacopsylla* species possessed the same *Carsonella* OTU in all individuals. The *Carsonella* sequence reads were present in individuals at up to 1.2%. Both *Diaphorina* species were dominated by ‘*Ca.* Profftella armatura*’* (up to 96.2% of reads), but it was not present in the *Cacopsylla* or *Cornopsylla* species (Fig. 3).

All *Ca*. *heterogena* possessed abundant Enterobacteriaceae from OTU 147629 (12.3–47.3%) and OTU12 (4.9–18.9%), which differed by 13 nucleotides; and *Cacopsylla* sp. harboured the same two OTUs, but OTU12 was more abundant (29.2–87.5%) (Fig. 1). All *Co. rotundiconis* individuals were dominated by two Enterobacteriaceae OTUs (OTU147629 at 63.5–67.4% abundance and OTU13 at 31.1–34.2% abundance), where the representative sequences for these OTUs differed by only a single nucleotide. For these symbionts classified to the Enterobacteriaceae, sequence searches using BLAST showed that the closest match to reference genomes in GenBank was *Buchnera aphidicola* Munson et al., but at only 91.6% match, these OTUs were not classified to genus and therefore, by convention, referred to as S-endosymbionts of its host. However, the sequences were closely related to other S-endosymbionts from other Psylloidae including other species of *Cacopsylla* (Fig. 3).

### Identity of other endosymbionts

**Fig. 4:**
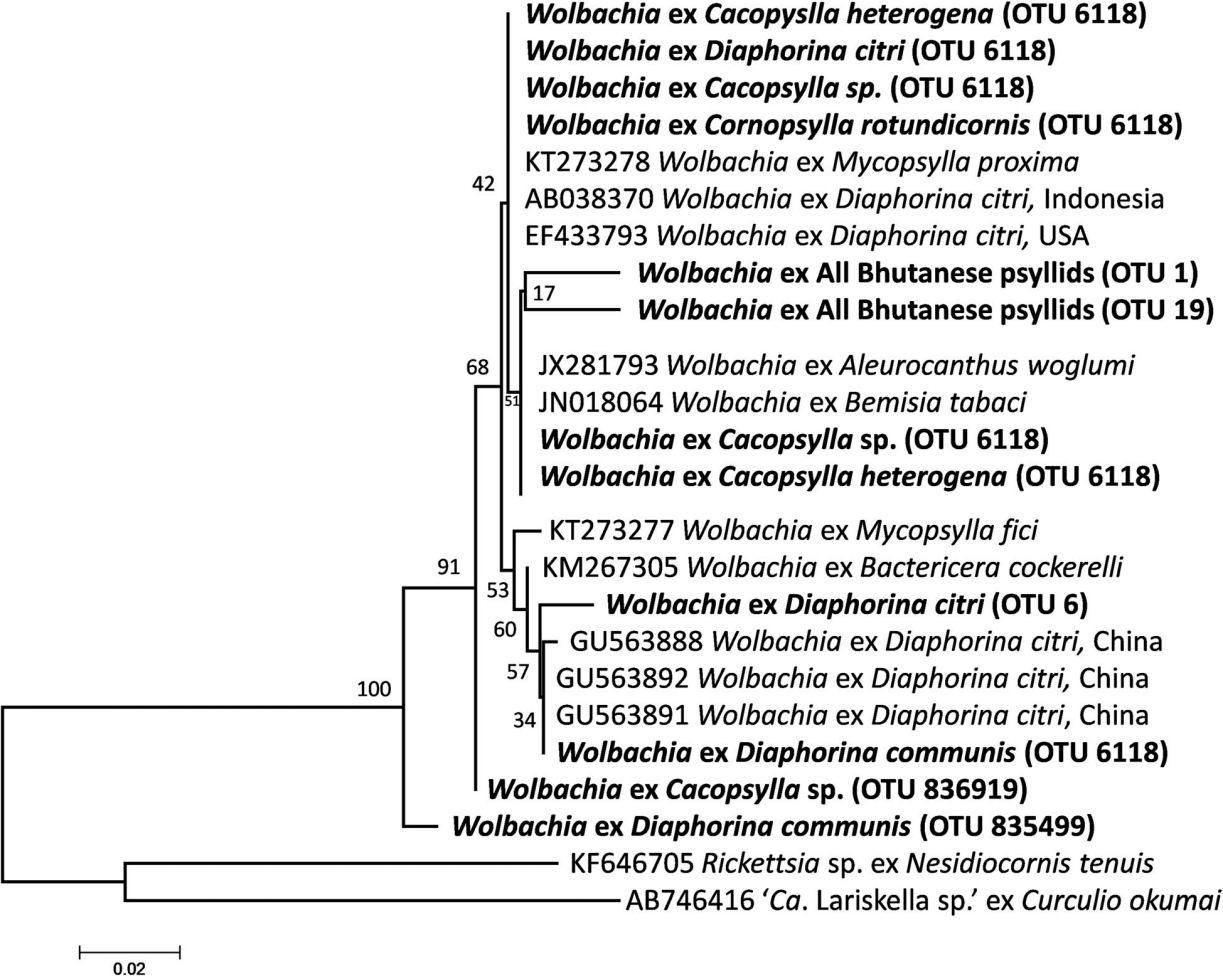
Phylogenetic analysis of 16S rRNA gene sequences of potential *Wolbachia* endosymbionts within the five species of Bhutanese psyllids (emboldened). The evolutionary history was inferred using maximum likelihood based on the Kimura 2-parameter model [54]. The tree with the highest log likelihood (− 1128.97) is shown. The percentage of trees in which the associated taxa clustered together is shown next to the branches. A discrete Gamma distribution was used to model evolutionary rate differences among sites (5 categories (+G, parameter = 0.3912)). The tree is drawn to scale, with branch lengths measured in the number of substitutions per site. The tree was rooted using accession AB746416. (NB OTU numbers classified according to Greengenes database; see Supplementary Table 3)

*Wolbachia* (Alphaproteobacteria: Rickettsiales) was prevalent in the five psyllid species (Figs. 1 & 4) at high titre in the *Diaphorina* and *Cacopsylla* species (in all individuals except a single *D. citri* male) but at low titre in all individuals of *Co. rotundiconis*. *Wolbachia* sequences from all five species were classified together into OTU 6118 from the Greengenes database; however, the representative sequence for this OTU was highly abundant only in *Ca*. *heterogena* and the unidentified *Cacopsylla* sp. This sequence was not identical to either of the highly prevalent *Wolbachia* strains previously reported in *D. citri*. Upon examination of the total reads, another sequence that differed by just one nucleotide, but still classified as OTU 6118, was found in *D. citri* samples as well as the *Cornopsylla* and *Cacopsylla* species, this sequence is identical to the *Wolbachia* sequences previously detected in *Diaphorina citri* from the USA. The *Wolbachia* strain from a Chinese isolate of *D. citri*, named *w*Di, was most abundant in *D. communis*, but this strain was not detected in the Bhutanese *D. citri* accessions screened here. These *Wolbachia* sequences were also confirmed in whole genome sequencing data derived from Bhutanese populations of *D. communis*, *Ca*. *heterogena* and the *Cacopsylla* sp. (data not shown). Other *Wolbachia* sequences were grouped into another five OTUs at low relative abundance levels (< 0.2% of reads) but the validity of these sequences could not be confirmed from whole genome sequence datasets.

**Fig. 5:**
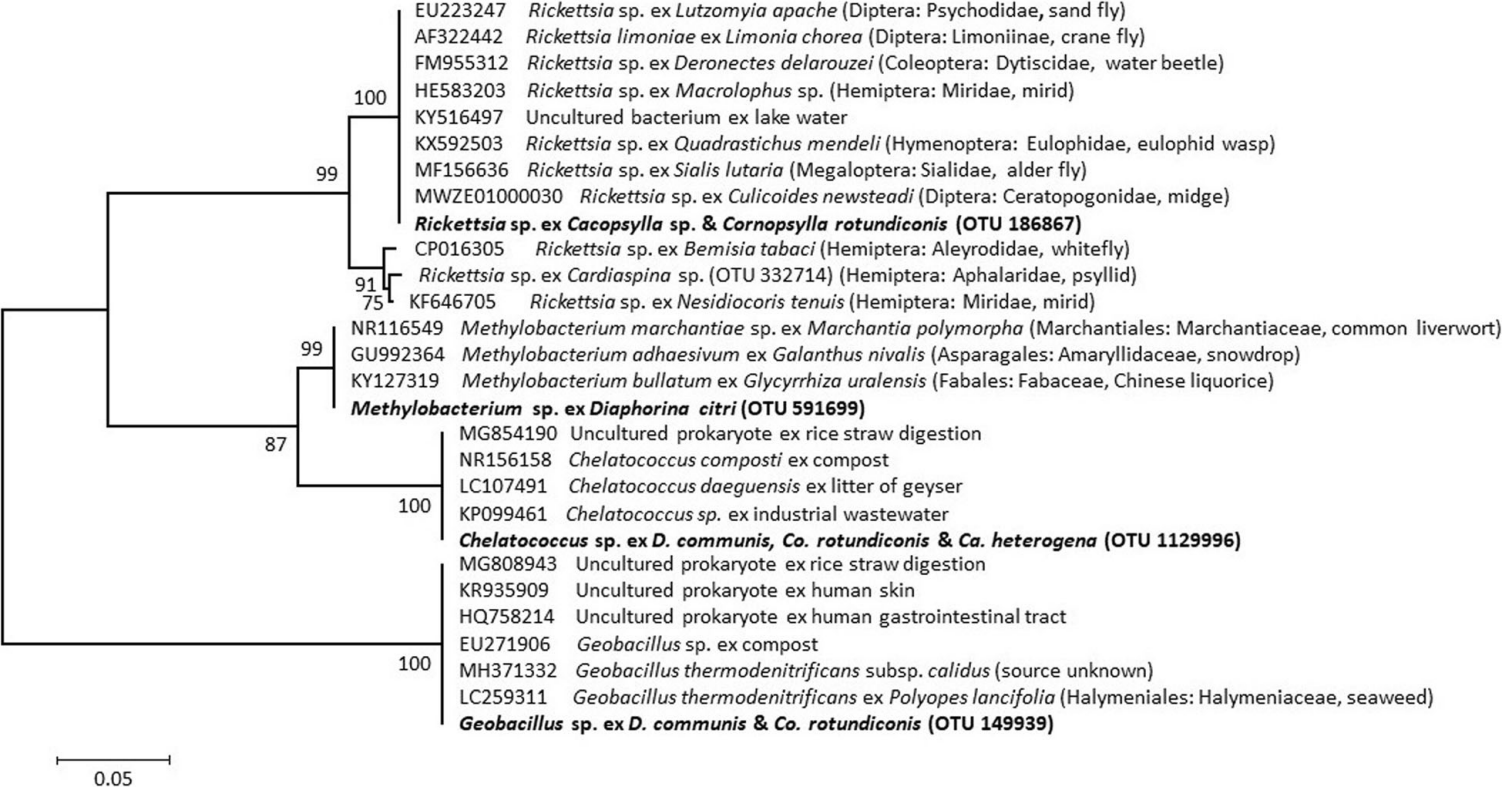
Phylogenetic analysis of 16S rRNA gene sequences of other bacteria found within the five species of Bhutanese psyllids (emboldened). The evolutionary history was inferred using maximum likelihood based on the Kimura 2-parameter model [54]. The tree with the highest log likelihood (− 1615.7) is shown. The percentage of trees in which the associated taxa clustered together is shown next to the branches. A discrete Gamma distribution was used to model evolutionary rate differences among sites (5 categories (+G, parameter = 0.6415)). The tree is drawn to scale, with branch lengths measured in the number of substitutions per site. The tree is unrooted. (NB OTU numbers are reference sequences in the Greengenes database; see Supplementary Table 3)

One *Rickettsia* (Alphaproteobacteria: Rickettsiales) OTU that displayed similarity to the endosymbionts of a range of insect species, as well as environmental sources, was found at high concentrations in certain *Co. rotundiconis* individuals, at low concentrations in other individuals of this species and at low concentrations in two individuals of the unidentified *Cacopsylla* sp. (Figs. 1 & 5).

Three individuals of the unidentified *Cacopsylla* sp. harboured a *Rickettsiella*-like OTU (Gammaproteobacteria: Legionellales) at up to 58% abundance and two individuals of the unidentified *Cacopsylla* sp. harboured the same OTU but at low concentrations, that showed high similarity to sequences of bacteria found in leaf hoppers and ixodid ticks, as well as other animal sources (Fig. S2).

The majority (6–9 out of 10) of individuals of *D. commumis* contained OTUs with similarity to *Chelatococcus* (Alphaproteobacteria: Rhizobiales); *Geobacillus* (Bacillis: Bacillales); *Niabella* (Sphingobacteria: Sphingobacteriales); *Meiothermus* (Deinococci: Thermales); *Buchnera* (Gammaproteobacteria: Enterobacteriales); and a member of the Comamonadaceae. These OTUs occurred at low abundance (0.02–0.77%). In addition, all individuals of *D. citri* harboured sequences of a single OTU at 0.6–3.8% abundance that shows high similarity to a range of *Methylobacterium* species (Alphaproteobacteria: Rhizobiales).

Individuals of both *Co. rotundiconis* and the unidentified *Cacopsylla* sp. contained OTUs with 100% identity with species of bacteria belonging in the genus *Agrobacterium* including *A. pusense*, *A. salinitolerans* and members of the *A. tumefaciens* complex*.* In addition, the majority of individuals (7 from 10) of the other species also contained low numbers of OTUs showing 100% identity with members of the genus *Staphylococcus*.

### Plant pathogens

**Fig. 6:**
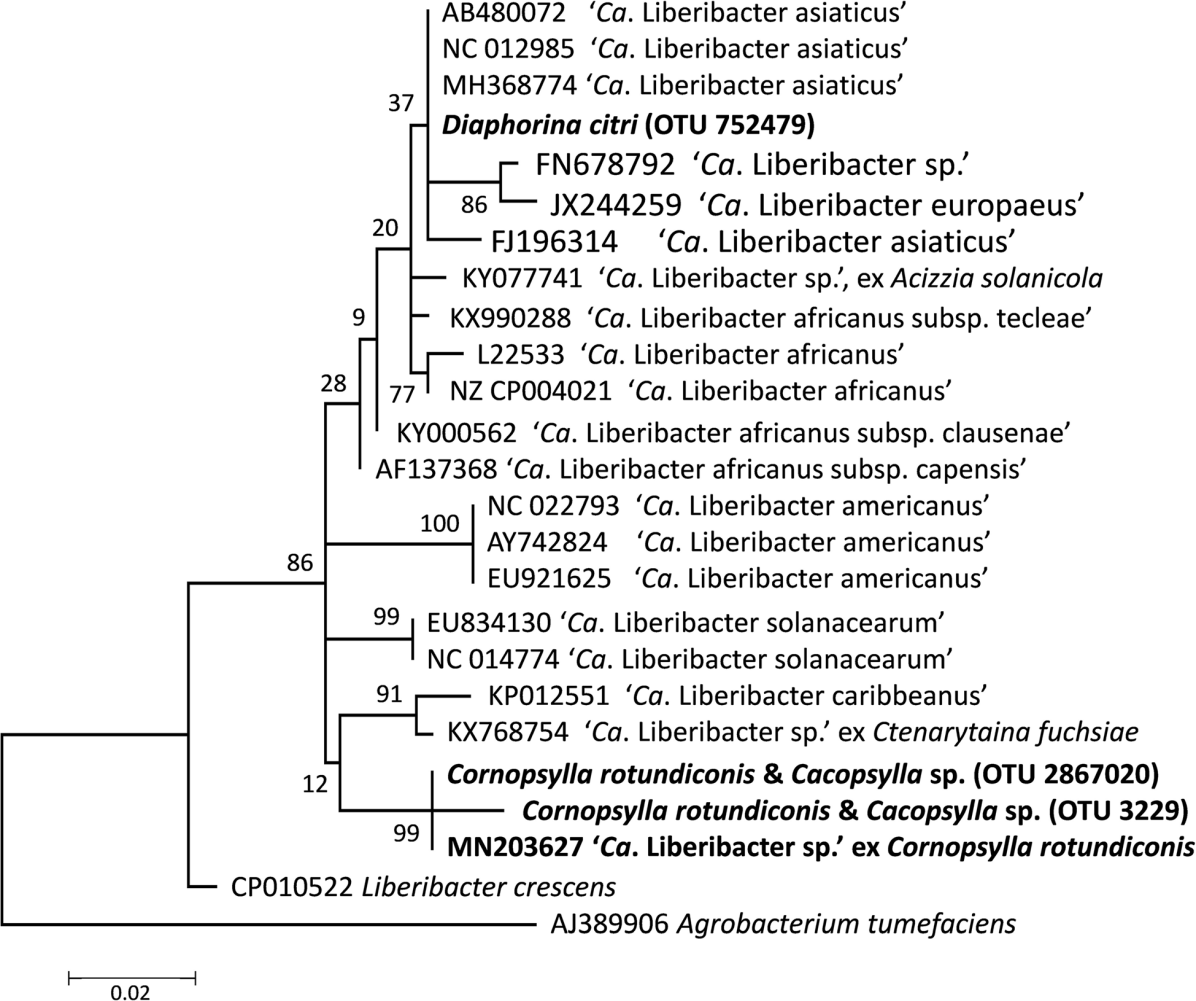
Phylogenetic analysis of 16S rRNA gene sequences of the ‘*Ca*. Liberibacter sp.’ amplified from *Cornopsylla rotundiconis* by PCR (GenBank accession MN203627) and from *Co. rotundiconis* and the unidentified *Cacopsylla* sp. by HTS (OTU numbers classified according to Greengenes database, also see Supplementary Table 3). The evolutionary history was inferred by using the maximum likelihood method based on the Kimura 2-parameter model [54]. The tree with the highest log likelihood (− 1039.25) is shown. A discrete Gamma distribution was used to model evolutionary rate differences among sites (5 categories (+*G*, parameter = 0.1544)). The tree is drawn to scale, with branch lengths measured in the number of substitutions per site. The tree is rooted with *Agrobacterium tumefaciens*

Nine of the 10 *D. citri* individuals tested using high throughput sequencing (HTS) contained OTUs with similarity to CLas found in other accessions of this psyllid species (Figs. 1 & 6). OTUs classified by the Greengenes data base as a species of ‘*Ca*. Liberibacter’ were detected in the *Cacospylla* and *Cornospylla* spp.; these sequences were not identical to other ‘*Ca*. Liberibacter’ sequences in GenBank. Five of the composite samples from *Co. rotundicornis* subjected to liberibacter-specific PCR and Sanger sequencing returned fragments of the expected sizes for one or both of the primer pairs. No differences in sequence were detected in the region of the 16S rRNA gene that overlaps between the HTS and specific-PCR data for OTU 2867020 and by only one base in OTU 3229. Phylogenetic analysis of the HTS and Sanger sequencing data (Fig. 6) showed that the bacterium did not group with any of the species of ‘*Ca*. Liberibacter’ found in GenBank and was sister to all accessions other than *Liberibacter crescens* Fagen et al.

### Bacterial community diversity

**Fig. 7:**
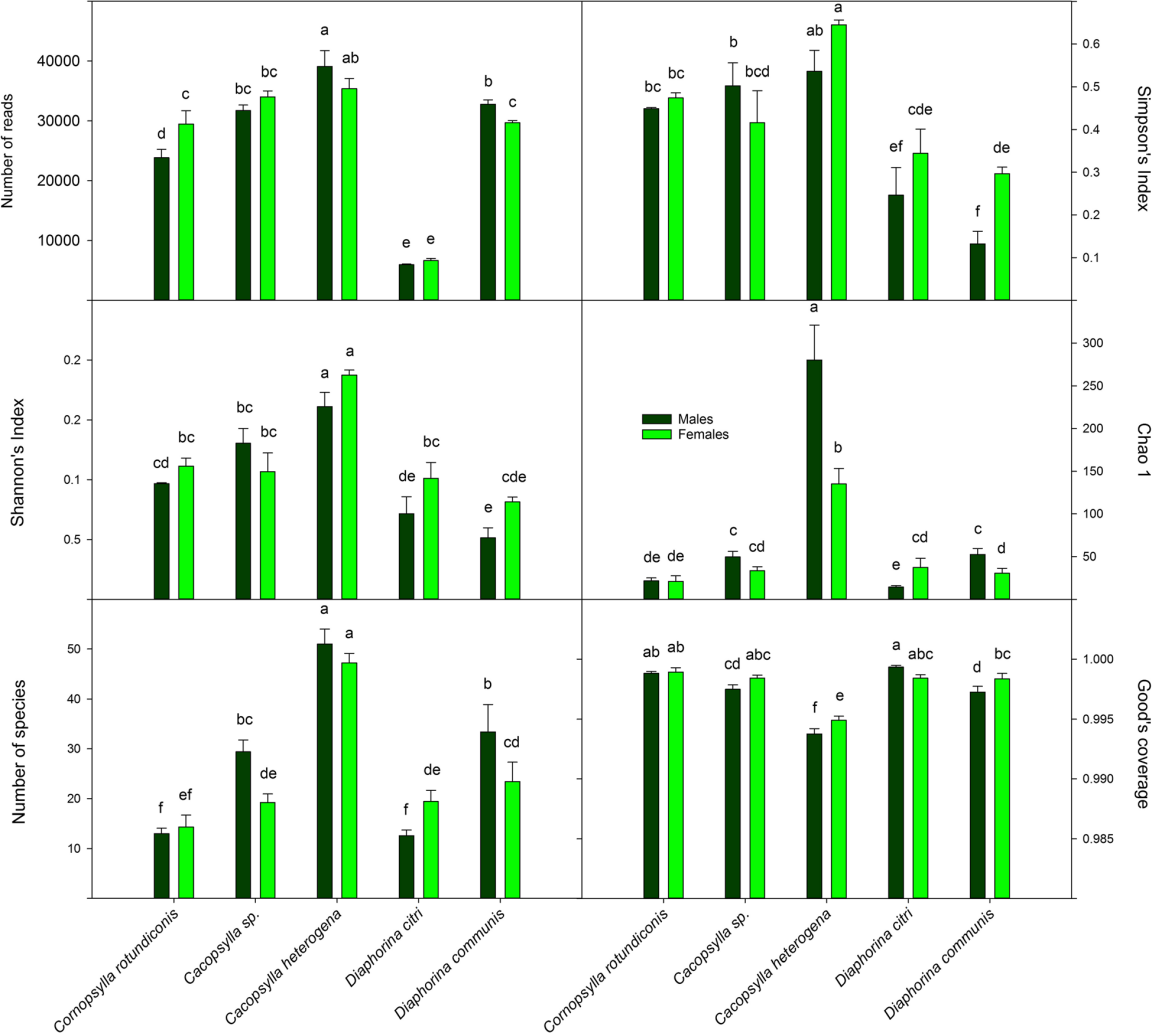
Measures of alpha-diversity, number of reads (A) Simpson’s (B), Shannon’s (C), Chao-1 (D), numbers of species (E) and Good’s (E) calculated from 16S rRNA gene amplicon sequencing of males and females of the five Bhutanese psyllid species. Error bars represent standard error of the means (*n* = 3–5). Means annotated with the same values are not significantly different from each other at *P* = 0.05 according to Student’s LSD tests

**Fig. 8:**
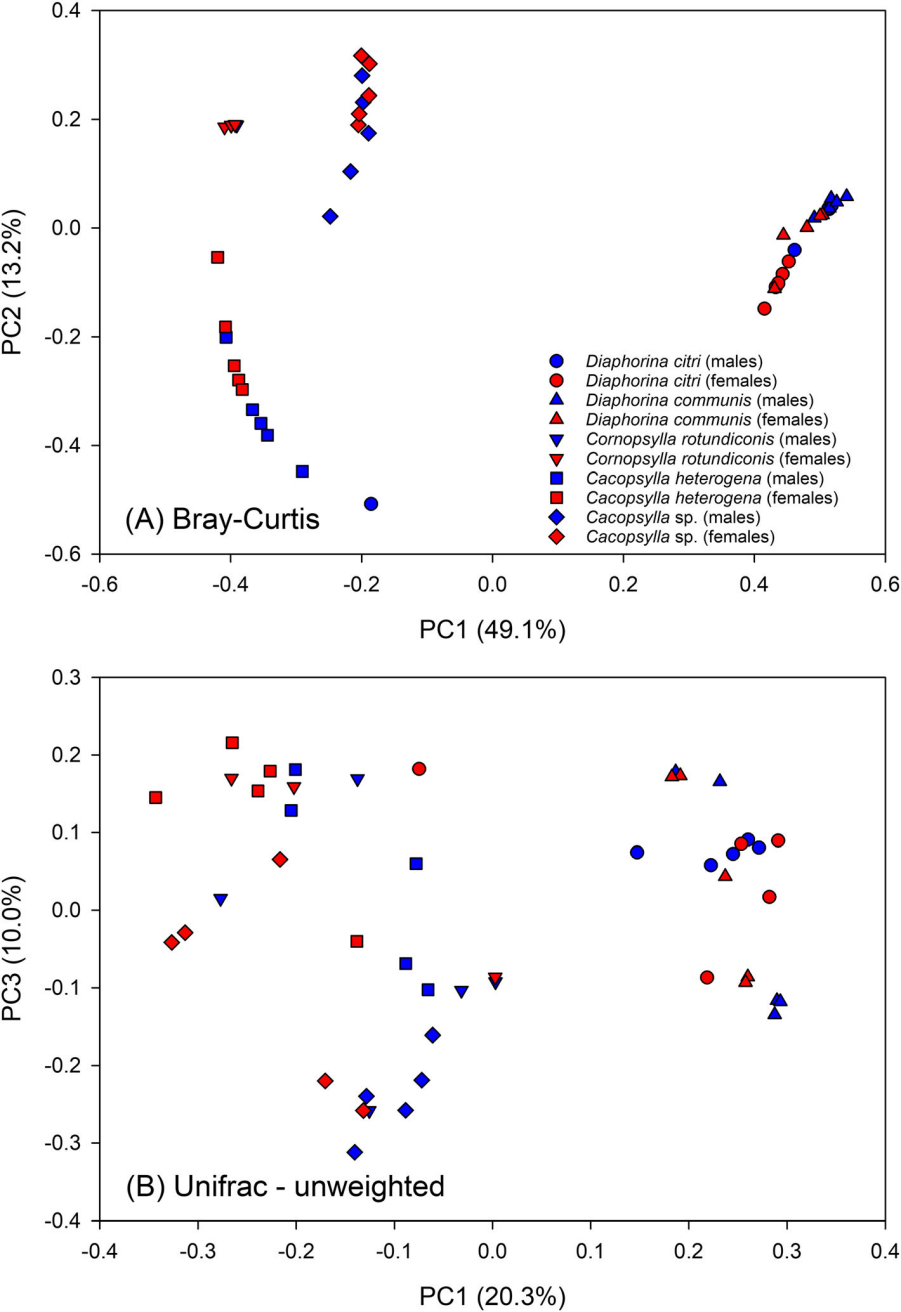
Principal coordinates analysis plot using **a** Bray-Curtis and **b** unweighted UniFrac distances generated from rarefied taxon abundances and depicting patterns of beta diversity among male and female psyllid of the five species found in Bhutan

Measures of α-diversity are presented in Fig. 7. As judged by both the number of species and the Chao1 metric, species richness appears to be greater in *Ca*. *heterogena* than in the remainder of the species. Both the Chao1 metric and Good’s Coverage suggest that this is due to a higher number of OTUs with few reads. The number of species in the other taxa are similar to each other. The measures of heterogeneity (Simpson’s and Shannon’s Indices) suggest that *Ca*. *heterogena* is most diverse and that the two species of *Diaphorina* are less diverse than the other three. Males of the undescribed species of *Cacopsylla* and *D. communis* carry more bacterial species than females whereas the opposite is true for *D. citri*; all effects of sex are, however, small. With the exception of the two species of *Diaphorina*, principal coordinates analysis (PCoA) coordination of measures of β-diversity (Fig. 8) separate the taxa from each other; the *Diaphorina* species group together, a result of the dominance of *Profftella* reads in both *Diaphorina* species. Small effects of sex are also apparent, especially for *D. citri* and *Ca*. *heterogena*.

## Discussion

Given the importance of endophytic bacteria to crop protection and to the biology of psyllids, this study has looked at the microbiomes of five species of psyllids associated with Rutaceae in Bhutan. As has been found for a range of plant sap-feeding insects [32, 55], the microbiomes of the psyllids largely consisted of their P- and S-endosymbionts, and *Wolbachia*. All five species contained OTUs related to *Carsonella*, the P-endosymbiont present in all psyllid species [33]. The P-endosymbionts from the insects in this study grouped with those from closely related taxa showing the strong co-phylogeny between insects and their P-endosymbionts[28]. *Cacopsylla heterogena* and *Cacopsylla* sp. share the same *Carsonella* OTU, suggesting these two psyllid species are closely related [32]. Although there is no strict co-speciation between psyllids and their S-endosymbionts due to loss and acquisition [32, 56], the S-endosymbionts from the five psyllids grouped with those from closely related taxa. The accessions of *D. citri* and *D. communis* both contained sequences related to ‘*Ca*. Profftella armatura’, the defensive endosymbiont of some psyllids characterised by Nakabachi et al. [57]. So far, *Diaphorina* is the only genus found to harbour bacteria from this genus, and more work on the genomic and ecological features of the *Profftella* species associated with *D. communis* is needed to determine if it plays a similar defensive role as in *D. citri*. Previously, members of the genus *Arsenophonus* [58] were found in *D. citri* [56] as well as other psyllids [32, 56]; however, it was not detected in populations of the two *Diaphorina* species examined in this study. *Arsenophonus* sequences were also absent from *D. citri* sampled from Florida [59]; often invasive species suffer loss of bacterial endosymbionts in a new environment, and these Bhutanese populations may exemplify this scenario [32, 60].

The other three psyllid species, including *Co. rotundiconis*, all contained OTUs classified as Enterobacteriaceae. These bacteria are not currently able to be identified to the species level, and they represent novel S-endosymbionts that are a hallmark of the psyllid microbiome [32, 35]. These psyllid-associated S-endosymbionts appear fixed, or almost fixed, across individuals of a species, and are found in high relative abundance within an individual. It is likely that they share the attributes of the S-endosymbionts described in other psyllids in prior studies [32, 35], that is: (1) intracellular and non-culturable; (2) vertically transmitted; (3) evolving towards obligatory symbiosis within host; and (4) small, reduced genome size due to shedding genes during evolution to intracellular and obligate lifestyle. However, genome assemblies of these bacteria will be necessary to detect the signatures of genome reduction, and further tests performed to determine tissue localization and physiological effects in hosts. In addition, six of the populations of *D. communis* contained a relatively low abundance of sequences related to *Buchnera aphidicola* (99.8% identity), the P-endosymbiont of aphids [61]. Although *Buchnera* has been found in insects other than aphids [62], *Buchnera* have also been found in the plant, *Solanum nigrum* L. (Solanales: Solanaceae) [53] and in samples of human origin [63] and may be part of the group of OTUs representing environmentally-acquired bacteria.

*Wolbachia* was found in all five psyllid species and was abundant in all except *Co. rotundiconis.* Similar strains that all clustered as OTU6118 were detected in all species. *Wolbachia* is commonly found in arthropod species [64], and studies have found *Wolbachia* infection is fixed in *D. citri* populations in Brazil [65] but with differences detected in titres per individual [66]. The bacterium is a facultative, cytoplasmic symbiont that manipulates reproduction in its host [67]. The presence of *Wolbachia* proteins in the saliva of *D. citri* indicates that *Wolbachia* may play a role in psyllid–plant interactions [68]. Two prevalent *Wolbachia* strains that have been reported in *D. citri*, were also detected in this dataset at high abundance, with the Bhutanese *D. citri* dominated by a single *Wolbachia* strain first found in USA isolates of *D. citri*, while *D. communis* samples were dominated by the *Wolbachia* strain, *w*Di, first detected in *D. citri* from China. Due to the importance of *D. citri* as a vector of liberibacter, and the apparent lower competence of *D. communis* to transmit liberibacters, contrasting the potential impact of *Wolbachia* in both *D. citri* and *D. communis* adults and nymphs may prove insightful. The abundant *Wolbachia* strains in the two *Cacopsylla* species were most closely related to those from species of whitefly.

The unidentified *Cacopsylla* sp. was infected with a species of *Rickettsia* showing sequence identity to accessions of this genus from a range of insect species; however, the sequences were diverged from *Rickettsia* found in some other psyllids and the white fly, *Bemisia tabaci* (Gennadius) (Hemiptera: Aleyrodidae) [32, 69, 70]. *Rickettsia* can be obligate, intracellular symbionts that are vertically transmitted but can also be acquired by whiteflies from a plant host inside which they become established in the phloem [71]. Like *Wolbachia*, some *Rickettsia* strains can affect host reproduction through male-killing [72] and parthenogenesis [73] and can also reduce microbiome diversity [74, 75]. They can also affect crop protection, as *Rickettsia* increase susceptibility to insecticides [76, 77], and Davis et al. [78] have shown that papaya bunchy top disease is caused by a *Rickettsia* vectored by *Empoasca* leafhoppers (Hemiptera: Cicadellidae). The role of this organism in *Cacopsylla* needs to be investigated.

CLas was found in *D. citri* but not in *D. communis*. The lack of CLas in *D. communis* is not surprising as, although the psyllid ‘feeds’ on *Citrus* spp. plus *Murraya elongata* A.DC. ex Hook. F. (Aurantieae), *B. koenigii* and *Zanthoxylum* spp., its development appears to be restricted to *B. koenigii* [44]; *B. koenigii* is not a host of CLas [44, 79]. The discovery of an unidentified ‘*Ca.* Liberibacter sp.’ in *Co. rotundiconis* and the unidentified *Cacopsylla* sp. parallels the discovery of ‘*Ca*. Liberibacter brunswickensis’ in *Acizzia solanicola* Kent and Taylor (Psyllidae) [80] and ‘*Ca*. Liberibacter ctenarytainae’ in *Ctenarytaina fuchsiae* (Maskell) (Aphalaridae) [81]. Both *Co. rotundiconis* and *Cacopsylla* sp. share a host plant that may enable acquisition of this liberibacter by both psyllids. Within *D. citri*, CLas is able to replicate and titres of this bacterium are relatively high [82]; however, in our study, the number of reads of the unidentified ‘*Ca*. Liberibacter sp.’ within individuals of *Co. rotundiconis* and the unidentified *Cacopsylla* sp. were low. This may suggest that the bacterium is unable to replicate and may have been passively acquired through feeding. Alternatively, the bacterium may replicate poorly within the psyllids. Therefore, the status of the new liberibacter as a plant pathogen, its transmission and its potential plant hosts are currently unknown.

The generic liberibacter primers used in this study proved useful to validate the liberibacter sequences obtained by HTS. Analysis of the primer pair 484F/803R in silico determined that they will amplify all known species and subspecies of liberibacter at the time they were designed. A single mismatch between the 803R reverse primer and the 16S rRNA sequence of ‘*Ca*. L. europaeus.’ did not prevent its amplification. Since their design, two new liberibacter species that have been identified also have a single mismatch in a noncritical region of the reverse primer. As with other generic primers, 484F/803R may occasionally amplify sequences from non-liberibacter species but the identity of the resulting amplicon is easily resolved by sequence analysis.

A *Rickettsiella*-like organism was detected in *Co. rotundiconis* and the unidentified *Cacopsylla* sp. This OTU (576671) has 99.5% sequence identity with *Diplorickettsia massiliensis* Mediannikov et al. (Legionellales: Coxiellaceae) from an *Ixodes* ticks and 98.6% identity from a *Diplorickettsia* sp. from a murine mucosa. Related organisms from the Coxiellaceae have been found in the psyllids, *Cecidotrioza sozanica* Boselli [83] and *Glycaspis brimblecombei* Moore [32]; however, these are more distantly related to OTU 576671. *D. massiliensis* is an obligate intracellular bacterium [84] and human pathogen [85] and is phylogenetically related to the genus *Rickettsiella*. Most *Rickettsiella* spp. are arthropod pathogens [86]; however, facultative symbionts belonging to the genus *Rickettsiella* have been identified in the aphid, *Acyrthosiphon pisum* Harris [87] and in the leafhoppers, *Orosius albicinctus* Distant [88], *Macrosteles striifrons* (Anufriev) and *Macrosteles sexnotatus* (Fallén) [89]. The *Rickettsiella* sp. within *A. pisum* causes changes in body color that, in turn, may influence predator-prey interactions [87], and any effects of OTU 576671 on the biology of its hosts need to be determined.

*Diaphorina citri* harboured bacteria belonging to the genus *Methylobacterium* that showed sequence homology with bacteria of this genus from a range of plant species. This xylem-associated bacterium has been isolated from citrus plants [90, 91], and it is possible that the psyllid acquired this bacterium during periods of xylem feeding. *Diaphorina communis* and to a lesser extent *Co. rotundiconis* and *Ca*. *heterogena* harboured a number of OTUs associated with environmental samples: *Geobacillus* and *Chelatococcus,* commonly found in straw products and composts; *Niabella* from soils; *Meiothermus* from biofilms from geothermal areas; *Thermomonas* from a fixed-bed reactor, hot springs, and kaolin slurry; and a member of the *Comamonadaceae,* other members of which are found ubiquitously in freshwater habitats and from activated sludge. Insect microbiomes can vary considerably in diversity with plant sap-feeding insects usually containing few gut bacteria [92, 93]. These bacteria in *Co. rotundiconis* may represent passive acquisition through consumption rather than active colonisation of the insect’s gut.

OTUs showing 100% identity with members of the *Staphylococcus* were found in the unidentified *Cacopsylla* sp. Staphylococci are commonly found in the microbiomes of insects [94] and have been shown to affect insect behaviour. For example, the velvetbean caterpillar (*Anticarsia gemmatalis* Hübner, Lepidoptera: Noctuidae) uses gut bacteria including staphylococci to overcome the activity of protease produced by its host plant [95] and the honey dew of *Acyrthosiphon pisum* contains a kairomone produced by *Staphylococcus sciuri* Kloos et al. (Bacillales: Staphylococcaceae) attracting ovipositing female hover flies (Syrphidae [96]). Further OTUs in the unidentified *Cacopsylla* sp. and in *Co. rotundiconis* showed 100% identity with members of the *A. tumefaciens* complex and with *A. pusense*. These bacteria have intimate associations with plants as gall-forming pathogens [97] or are involved in nitrogen fixation [98]. Further work is also required to determine whether the OTUs related to staphylococci and agrobacteria have biological roles associated with the psyllids or are again present due to consumption.

## Conclusions

The bacterial microbiota detected in these Bhutanese psyllids support the trends that have been seen in previous studies: psyllids have microbiomes largely comprising their obligate P-endosymbiont *Carsonella*, and one or two S-endosymbionts that are fixed and specific to each lineage. In addition, the association with plant pathogens has been demonstrated, with the detection of liberibacters in a known host, *D. citri*, and identification of a putative new species of liberibacter in *Co. rotundiconis* and *Cacopsylla* sp. Further molecular and biological characterisation of the new liberibacter species will provide additional insights on the potential environmental and/or economic impacts of this organism. The significance of liberibacters to global horticultural industries will likely prompt testing to determine the distribution of the putative species detected in this study outside of Bhutan and its presence in other hosts.

## Methods

### Psyllid collection and characterisation

Five species of three genera within the Psylloidea were sampled from host plants in Bhutan (Table 1, Supplementary Table 2). *Diaphorina citri* was first described from adults found on citrus in Taiwan. *Diaphorina communis* was first recorded by Mathur [99] on curry leaf (*Bergera koenigii* L.) (Clauseneae) in the northern Indian foothills of the Himalayas, but was not described until 1975 [100]. In Bhutan, it is found on both curry leaf and mandarin (*Citrus reticulata* Blanco) (Aurantieae) at elevations ranging from 210 to 1223 m asl. *Cacopsylla heterogena* is closely related to *Ca*. *citrisuga* (Yang & Li); in Bhutan, it was found on mandarin at elevations 983–2444 m asl [44] and in China is known to occur on citrus [46, 101]. *Cacopsylla* sp. and *Co. rotundiconis* both feed on *Zanthoxylum* spp*.* (Amyridoideae).

**Table 1:** Host plants and collection sites of the psyllid species used for high throughput sequencing

Species	Plant host	Collection date	Place
*Diaphorina communis*	*Bergera koenigii*	8/5/2014	Basochhu (27.3646 N; 89.9124 E)
*Cacopsylla heterogena*	Mandarin	19/4/2015	Riserboo A (26.9532 N, 90.1347 E)
*Cacopsylla* sp.	*Zanthoxylum* sp.	22/5/2014	Dangreyboog (27.0120 N; 90.1579 E)
*Cornopsylla rotundicorni*s	*Zanthoxylum* sp.	22/5/2014	Dangreyboog (27.0120 N; 90.1579 E)
*Diaphorina citri*	Mandarin	28/5/2013	Gosarling (27.0333°N, 90.1126°E)
*Diaphorina citri*	Mandarin	7/6/2013	Gosarling (27.0333°N, 90.1126°E)
*Diaphorina citri*	Mandarin	5/7/2013	Meme (27.0393°N, 90.1110°E)

Adult specimens for each species were used in high throughput bacterial microbiome analysis. Psyllids were collected by tapping branches of host plants onto a white enamel tray and aspirating the adults falling on the tray into a glass vial; the insects were then stored in absolute ethanol. For each species, the surfaces of five male and five female individuals were disinfested using 4% sodium hypochlorite as described in Morrow et al. [32] before DNA was extracted from whole, individual psyllids using QiaAmp Mini DNA Extraction kit (Qiagen) including an RNase treatment with 0.4 mg RNase (Sigma). The quantity and quality of the DNA was assessed by Nanodrop spectrophotometry, Qubit 2.0 Fluorometry and gel electrophoresis; all DNA samples were subject to a test amplification using general eubacterial 16S rRNA gene primers (63F and 1227R [102, 103]; Supplementary Table 1). DNA extracts were submitted to the HIE Next Generation Sequencing Facility (Hawkesbury Institute for the Environment, Western Sydney University) for HTS of the 16S rRNA gene using primers 341F and 805R, which cover the V3-V4 region of the 16S rRNA gene and produce a fragment of approximately 464 bp. Libraries were prepared using the Nextera XT kit (Illumina) with 7 ng of template DNA, and 2 × 300 bp paired end sequencing was run on a 384-multiplexed Illumina MiSeq lane.

### Data analysis using QIIME

Raw sequencing reads (fastq format) from 48 individual libraries were examined using FastQC v0.11.4 (http://www.bioinformatics.babraham.ac.uk/projects/fastqc). To increase the accuracy in these libraries [104], they were trimmed, then primer sequences were removed and paired reads joined as detailed in Morrow et al. [32].

QIIME 1.8.0 [105] was used to classify and analyse the bacterial communities associated with each sample using the procedures detailed in Morrow et al. [32]. Sequences were clustered into operational taxonomic units (OTUs) at 97% sequence similarity, and using uclust [106], classified according to the Greengenes 13_8-release database; OTUs comprising fewer than three sequences were removed. Chimeras were identified using the blast_fragments method [107] and then removed. Rarefaction graphs were generated in the vegan package within the R environment [108] to determine the optimal number of sequences for normalisation of samples. Metrics of alpha (Chao1, Shannon and Simpson indices and Good’s coverage) and beta diversity (Bray-Curtis similarities and weighted and unweighted UniFrac) were also determined as detailed in Morrow et al. [32]. Principal coordinate analyses were performed using the vegan package from within the R environment. Statistical tests of alpha and beta diversity comparisons by category (species, genus and sex) were performed using Statistica (Version 13.3; TIBCO Software Inc.).

### 16S rRNA gene phylogeny

DNA sequence alignments of 16S rRNA genes derived from the HTS and sequences sourced from GenBank were produced using Muscle, as implemented in MEGA7 (Version 7.0.26 [109]). Prior to analyses, the most appropriate evolutionary model for each data set was selected based on their Bayesian Information Criterion and phylogenetic relationships estimated using maximum likelihood. The percentages of trees in which the associated taxa clustered together were determined from 1000 bootstrap replications.

To confirm the novel liberibacter sequences discovered in the HTS data, DNA was extracted from 10 composite samples of five *Co. rotundiconis* adults. Almost the entire (1411 bp) 16S rRNA gene was amplified by PCR using the generic liberibacter primer pairs LG774F/LG1463R [80], 484F/1124R (Lia Liefting pers. comm.) and fD2 [110] /803R (Lia Liefting pers. comm.) (See Supplementary Table 1 for primer information). The fragments obtained were subjected to Sanger sequencing, and the data assembled and aligned using Geneious (Version 10; Biomatters Ltd.) together with relevant sequences from GenBank. The aligned data were subjected to phylogenetic analysis using MEGA7 as described above. The tree obtained was rooted using a sequence from *Agrobacterium tumefaciens* Smith & Townsend (Alphaproteobacteria: Rhizobiales)*.* The sequence from *Co. rotundiconis* was submitted to GenBank (MN203627).

## Supplementary information

**Additional file 1: Figure S1.** Rarefaction curve of 16S rRNA gene sequences from male and female psyllids. Curves were calculated based on operational taxonomic units (OTUs) at 97% similarity.

**Additional file 2: Figure S2.** Phylogenetic analysis of 16S rRNA gene sequences of Rickettsiella-like bacteria found within two of the species of Bhutanese psyllids (emboldened). The evolutionary history was inferred using maximum likelihood based on the Kimura 2-parameter model [54]. The tree with the highest log likelihood (− 1263.03) is shown. A discrete Gamma distribution was used to model evolutionary rate differences among sites (5 categories (+G, parameter = 0.3116)). The rate variation model allowed for some sites to be evolutionarily invariable ([+I], 55.45% sites). The tree is drawn to scale, with branch lengths measured in the number of substitutions per site. (NB OTU numbers are reference sequences in the Greengenes database; see Supplementary Table 3 and [30]).

**Additional file 3: Supplementary Table 1.** Primer sequences and PCR conditions.

**Additional file 4: Supplementary Table 2.** Sample metadata and alpha diversity metrics.

**Additional file 5: Supplementary Table 3.** OTU name and abundance based on the 16S rRNA amplicon dataset rarefied to 5612 sequences, for each of the 48 psyllid samples.

**Additional file 6: Supplementary Table 4.** Representative set of 16S rRNA gene sequences of OTUs from five psyllid species from Bhutan.

## Data Availability

The raw 16S rRNA genes amplicon reads are available from NCBI, SRA BioProject ID PRJNA563783. The assembled OTU representative sequences are found in fasta format in Supplementary Table S4. The DNA sequence for the Liberibacter sp. ex *Cornopsylla rotundiconis* 16S rRNA gene is available from GenBank, accession number MN203627.

## Abbreviations

OTU: Operational taxonomic unit
CLas: ‘*Ca.* Liberibacter asiaticus’
P-endosymbiont: Primary endosymbionts
S-endosymbiont: Secondary endosymbiont
HTS: High throughput sequencing
PCR: Polymerase chain reaction
PCoA: Principal coordinates analysis
bp: Base pair
