# Supplementary figures and images for: Characterization of the bacterial communities of psyllids associated with Rutaceae in Bhutan by high throughput sequencing
Source: BMC Microbiol. 2020 Jul 20;20:215. doi: 10.1186/s12866-020-01895-4 (PMC7370496; doi:10.1186/s12866-020-01895-4)

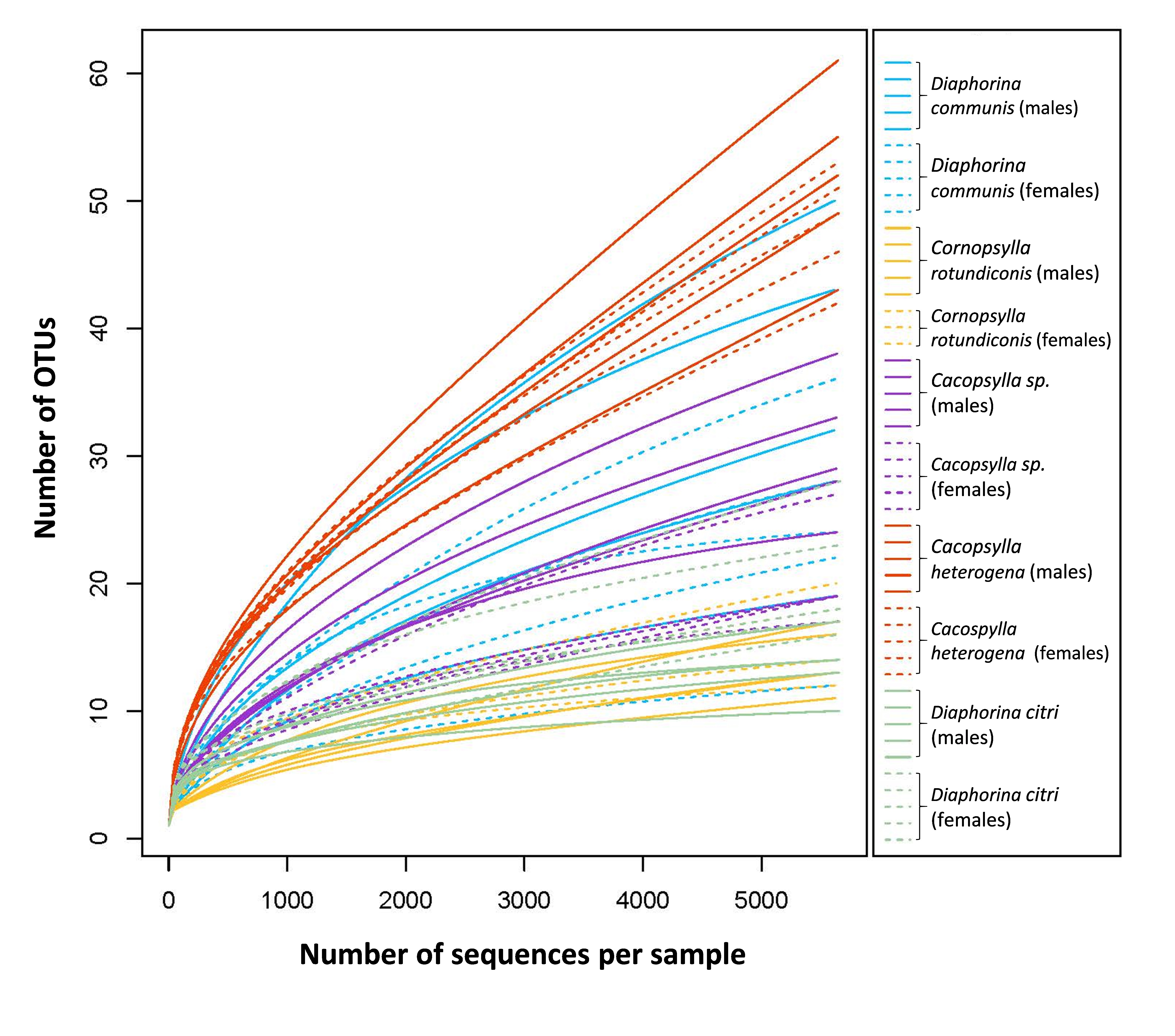

Supplement: Supplementary file 1 — Additional file 1: Figure S1. Rarefaction curve of 16S rRNA gene sequences from male and female psyllids. Curves were calculated based on operational taxonomic units (OTUs) at 97% similarity. [file 12866_2020_1895_MOESM1_ESM.jpg]

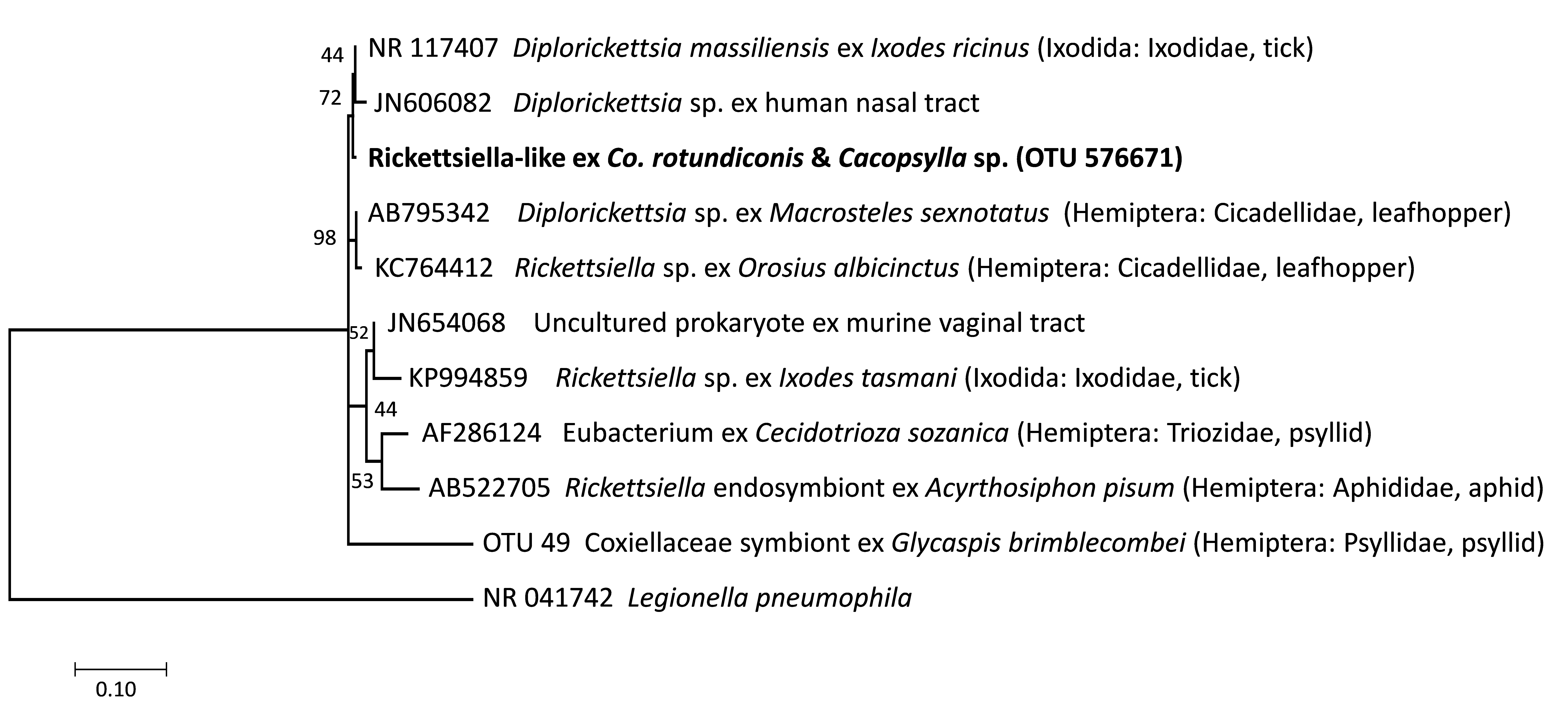

Supplement: Supplementary file 2 — Additional file 2: Figure S2. Phylogenetic analysis of 16S rRNA gene sequences of Rickettsiella-like bacteria found within two of the species of Bhutanese psyllids (emboldened). The evolutionary history was inferred using maximum likelihood based on the Kimura 2-parameter model [54]. The tree with the highest log likelihood (− 1263.03) is shown. A discrete Gamma distribution was used to model evolutionary rate differences among sites (5 categories (+G, parameter = 0.3116)). The rate variation model allowed for some sites to be evolutionarily invariable ([+I], 55.45% sites). The tree is drawn to scale, with branch lengths measured in the number of substitutions per site. (NB OTU numbers are reference sequences in the Greengenes database; see Supplementary Table 3 and [30]). [file 12866_2020_1895_MOESM2_ESM.jpg]
